# Glycosylation profile of *Mycobacterium leprae*-specific antibodies associated with disseminated infection

**DOI:** 10.1016/j.isci.2026.116373

**Published:** 2026-06-20

**Authors:** Anouk van Hooij, Wenjun Wang, Cristiana Santos de Macedo, Jan Nouta, Steinar Gijze, Roberta Olmo Pinheiro, Colette L.M. van Hees, Manfred Wuhrer, Annemieke Geluk

**Affiliations:** 1 Department Leiden Center of Infectious Diseases, LUCID, Leiden University Medical Center, Leiden, the Netherlands; 2 Center for Proteomics and Metabolomics, Leiden University Medical Center, Leiden, the Netherlands; 3 Center for Technological Development in Health, Fiocruz, Rio de Janeiro, Brazil; 4 Leprosy Laboratory, Oswaldo Cruz Institute, Fiocruz, Rio de Janeiro, Brazil; 5 Department of Dermatology, Erasmus Medical Center, Rotterdam, the Netherlands

**Keywords:** biological science

## Abstract

Leprosy still affects hundreds of thousands of new patients each year and can result in permanent nerve damage if not treated timely. *Mycobacterium leprae*-specific antibodies directed against the major cell-wall component, phenolic glycolipid-I (PGL-I), are associated with disseminated infection, suggesting that these antibodies do not provide adequate protection. Despite widespread use in diagnostic applications, the exact function of anti-PGL-I antibodies remains unknown. Therefore, antibody subclass distribution and Fc glycosylation of total and PGL-I-specific IgG were assessed by liquid chromatography-mass spectrometry, including leprosy patients from outpatient clinics in the Netherlands (*n* = 160) and Brazil (*n* = 55). The Fc domain of PGL-I-specific IgG1, the predominant subclass observed, showed elevated fucosylation, in combination with reduced sialylation and galactosylation. This distinctive glycoprofile of PGL-I specific IgG is generally linked to dampened effector functions, and points toward poor activation of effector mechanisms by these *M. leprae-*specific antibodies associated with disseminated infection.

## Highlights

• Patients with disseminated leprosy induced PGL-I-specific IgM and IgG
• IgG1 was the predominant subclass of *M. leprae*-specific PGL-I IgG
• Similar IgG1 Fc glycoprofiles were detected in Dutch and Brazilian patients
• PGL-I IgG1 was highly fucosylated, with lower sialylation and galactosylation

## Graphical abstract

**Figure:**
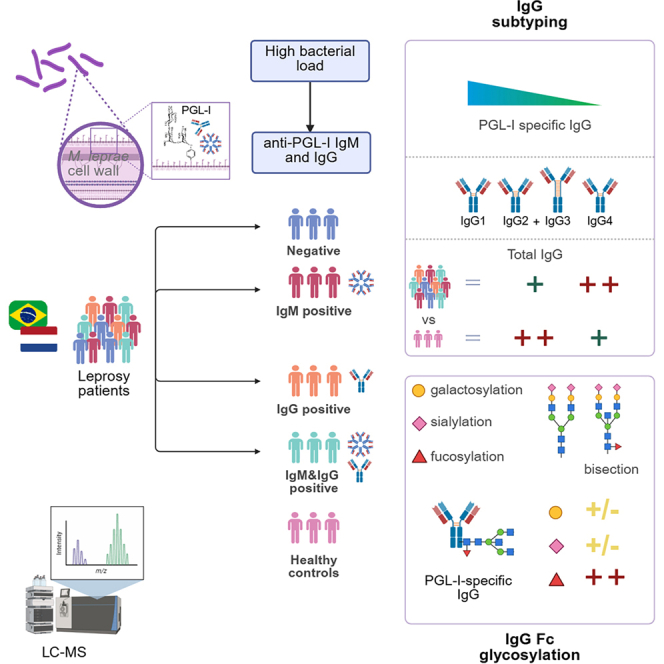

## Introduction

Leprosy is a chronic infectious disease, which mainly affects the skin and peripheral nerves. Although this poverty-related disease is endemic primarily in low- and middle-income countries, it is also on the rise in the Southeastern United States^1^^,^^2^^,^^3^ and autochthonous cases are being reported in Europe.^4^ If treatment is not initiated timely, infection of Schwann cells by *Mycobacterium leprae* can result in severe irreversible nerve damage, causing visible deformities.^5^ The disease presents as a clinical spectrum determined by host immunity to *M. leprae*, ranging from disseminated infection to few detectable bacilli contained in granulomas.^6^ Disseminated infection is characterized by a humoral immune response against *M. leprae*, leading to the production of numerous antibodies.^7^ This paradox of high antibody levels in the most severe forms of disease has also been described for COVID-19.^8^

The most widely studied are the antibodies directed against the major cell-wall component of *M. leprae*, phenolic glycolipid-I (PGL-I).^9^^,^^10^^,^^11^ PGL-I is responsible for the neural tropism of *M. leprae*, allowing the invasion of Schwann cells via binding of the specific trisaccharide part of PGL-I to the laminin α2 chain on the Schwann cell membrane.^5^ Moreover, this *M. leprae*-specific PGL is an important virulence factor as it enhances mycobacterial infectivity and can subvert host immunity, by inhibiting the production of inflammatory cytokines.^12^ Various diagnostic applications have implemented PGL-I-specific antibodies, sensitively and specifically detecting patients with high bacillary loads.^9^^,^^10^^,^^13^ Given the correlation between anti-PGL-I antibodies and bacterial load,^13^^,^^14^ it is improbable that these antibodies convey protective immunity against *M. leprae.* The exact role of these antibodies in leprosy disease, however, remains largely unknown.

The role of antibody-mediated immunity in mycobacterial disease has been ignored for decades due to the historical dogma that host defense against intracellular pathogens is mediated by cell-mediated immunity, rather than humoral immunity.^15^ Significant contributions of humoral immunity were reported, though, in individuals latently infected with another mycobacterium, *M. tuberculosis*, showing enhanced antibody effector functions to mycobacterial antigens compared to individuals with active tuberculosis disease.^16^ These results indicate that humoral immunity does contribute to effective mycobacterial killing.

Antibody effector functions are determined by isotype and subclass, which can be switched via antigen-driven class-switch recombination.^17^ Effector functions are also influenced by glycosylation of the fragment crystallizable (Fc) domain, modulating the activation of Fcγ receptors and complement pathways.^18^^,^^19^ Glycosylation is mainly unexplored in leprosy research: glycosylation of PGL-I-specific antibodies has not been studied previously, and reports on total IgG glycosylation in leprosy are scarce and dated.^20^^,^^21^ Skewing of IgG glycosylation toward specific patterns has been observed in other infectious diseases, both bacterial and viral,^22^^,^^23^ including tuberculosis.^24^^,^^25^ Different glycan modifications confer distinct effector functions; low fucosylation enhances natural killer (NK) cell-mediated antibody-dependent cellular cytotoxicity via increased FcγRIIIa and FcγRIIIb binding, and this effect is further enhanced by simultaneous high galactosylation. High galactosylation and sialylation also increase C1q binding of antibodies and further downstream complement deposition.^19^ Glycan profiling can thus provide detailed information on the effector functions of antibodies.

To gain insight into these antibody effector functions in leprosy pathology, this study determined IgG glycosylation patterns of total and *M.leprae*-specific (anti-PGL-I) antibodies. First, sera from leprosy patients were screened for the presence of PGL-I-specific antibodies and their respective isotypes. Secondly, patient samples were selected for the determination of total and anti-PGL-I IgG N-glycan profiles. These glycoprofiles provide insight into the effector functions of antibodies in leprosy patients, studying antibody responses across the disease spectrum, as well as during treatment. To evaluate the replicability of the identified profiles, two independent cohorts were analyzed—originating from outpatient clinics in the Netherlands and Brazil.

## Results

### Isotype distribution of anti-PGL-I-specific antibodies

**Figure 1:**
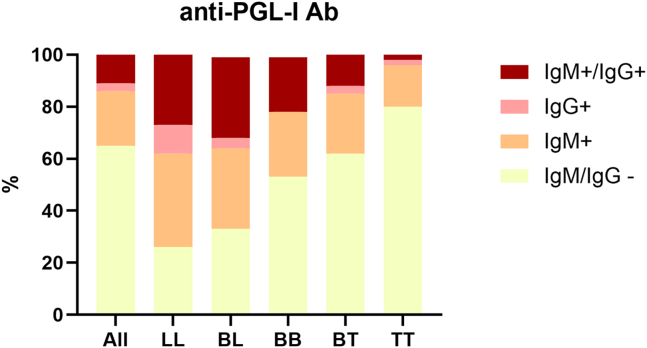
Distribution of anti-PGL-I IgM and IgG in leprosy patients Sera of 831 leprosy patients, obtained from patients at their first visit to the clinic in the Netherlands, were screened for the presence of PGL-I-specific antibodies to determine the isotype distribution. The percentage of seronegative patients (IgM-/IgG-; yellow), anti-PGL-I IgM positive (IgM+; orange), anti-PGL-I IgG positive (IgG+; pink); and anti-PGL-I IgM and IgG double-positive (IgM+/IgG+; red) is indicated on the *y* axis. Results are shown for all patients (All; *n* = 831) and patients stratified by classification: lepromatous (LL; *n* = 45), borderline lepromatous (BL; *n* = 96), midborderline (BB; *n* = 24), borderline tuberculoid (BT; *n* = 113), and tuberculoid leprosy (TT; *n* = 131).

Sera of 831 leprosy patients, obtained from patients at their first visit to an outpatient clinic in the Netherlands, were screened for the presence of PGL-I-specific antibodies to determine the isotype distribution. Irrespective of their diagnostic classification (Table S1), anti-PGL-I antibodies were detected in 35% of patients (Figure 1). IgM was more frequently observed than IgG, with 21% of patients presenting with anti-PGL-I IgM only at intake and 3% with anti-PGL-I IgG only. Anti-PGL-I IgM and IgG were simultaneously detected in 11% of patients. Stratification by classification indeed confirmed the predominant antibody response in lepromatous leprosy (LL)/borderline lepromatous (BL) patients with disseminated infection (Figure 1).

Subgroups of 40 age- and gender-matched patients were selected from the Dutch cohort based on the presence of PGL-I isotype to determine total and PGL-I-specific IgG subclass distribution and glycosylation patterns (Figure S1). Anti-PGL-I IgA was also assessed in these patient subgroups, and was detected mainly in patients presenting both anti-PGL-I IgM and IgG antibodies (Figure S1).

### Total IgG subclass distribution in leprosy patients and controls

**Figure 2:**
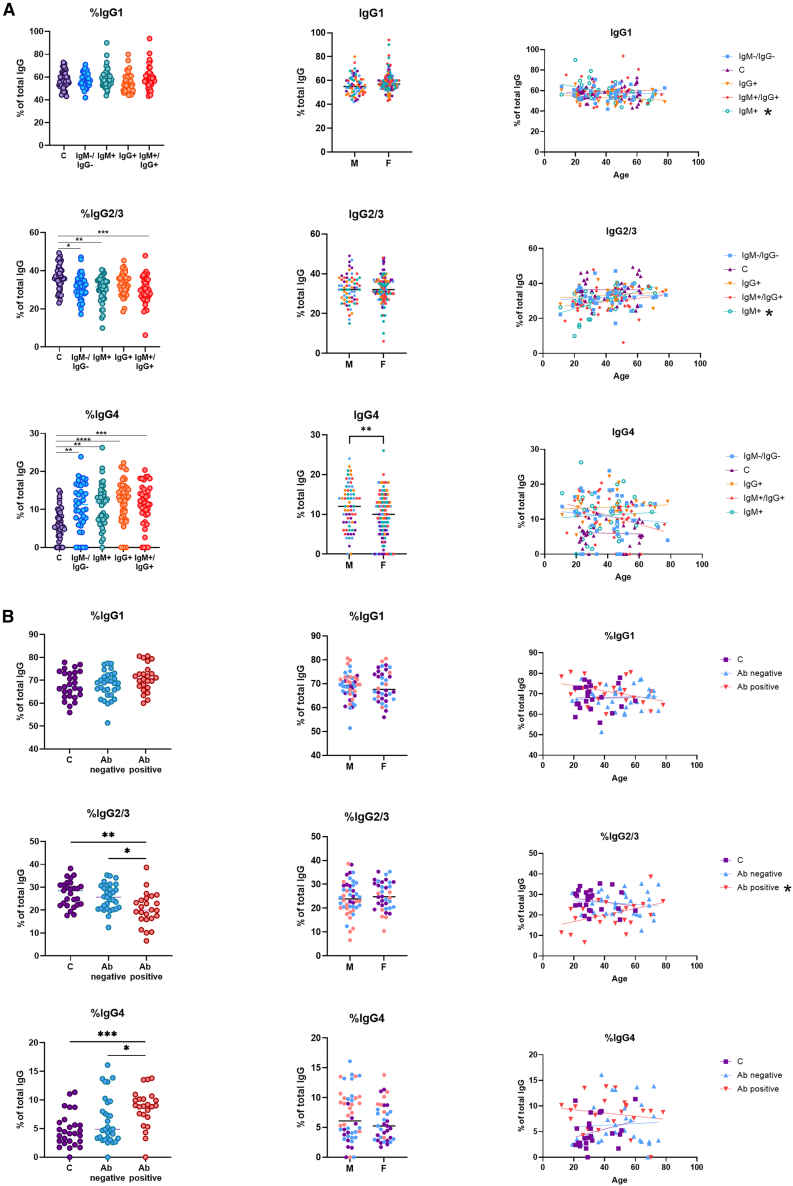
Total IgG1, IgG2/3, and IgG4 distribution in leprosy patients and controls The percentage of IgG1, IgG2/3, and IgG4 was determined by dividing the absolute intensity obtained by LC-MS of a specific subclass (i.e., IgG1) by the absolute intensity of the sum of all subclasses (IgG1 + IgG2/3 + IgG4) in patients originating from a Dutch (A) and Brazilian (B) outpatient clinic. Left column: The %IgG subclasses were compared between groups (scatterplot; line at median) by Kruskal-Wallis with Dunn’s correction for multiple testing. *p* value: ∗*p* < 0.05; ∗*p* < 0.01; ∗∗∗*p* < 0.001; ∗∗∗∗*p* < 0.0001. Groups Dutch cohort (*x* axis): control group (C), seronegative (IgM-/IgG-), IgM positive only (IgM+), IgG positive only (IgG+), or double-positive (IgM+/IgG-). Forty individuals were included per group. Groups Brazilian cohort: control group (C; *n* = 27), seronegative group (Ab negative; *n* = 31), seropositive group (Ab positive; *n* = 24). Middle column: %IgG subclass stratified by gender. The Mann-Whitney U test was performed to compare males (M) and females (F). Groups are indicated by color according to the legend. Right column: Pearson correlation between age and %IgG subclasses was determined, and simple linear regression lines were computed. Symbol and line colors correspond to the five groups as indicated in the legend. ∗ Indicates a significant correlation for this particular group.

IgG can be further divided into subclasses IgG1-4, each with specific effector functions. Therefore, IgG subclasses were determined by mass spectrometry for the four subgroups (*n* = 40) based on PGL-I isotype presence (IgM-/IgG-; IgM+; IgG+; IgM+/IgG+), as well as a healthy control group (*n* = 40; Figure S1; Table S2). All four patient groups showed lower percentages of total IgG2/3 and higher percentages of total IgG4 compared to healthy controls (Figure 2A). An age effect was observed within the IgM+ group, displaying a weak correlation with %IgG1 (R^2^ = 0.21) and %IgG2/3 (R^2^ = 0.27). This age effect was not observed in the other four groups. The %IgG4 was influenced by gender, being significantly lower in females compared to males (*p* < 0.0093). However, when analyzed separately by sex, IgG4 remained significantly higher in patients compared to controls (males: *p* = 0.0006; females: *p* < 0.0001). Total IgG subclass distribution is, thus, altered considerably in leprosy patients presenting to an outpatient clinic in a non-endemic region. In patients (*n* = 55) diagnosed in a Brazilian outpatient clinic, the country with the second-highest leprosy burden worldwide, decreased total %IgG2/3 and increased total %IgG4 compared to healthy controls (*n* = 27) was also observed in patients presenting with anti-PGL-I antibodies (Figure 2B; Table S3). In both cohorts, the subclass distribution of total IgG was not associated with leprosy classification (Figure S2).

### PGL-I-specific subclass distribution in leprosy patients and controls

**Figure 3:**
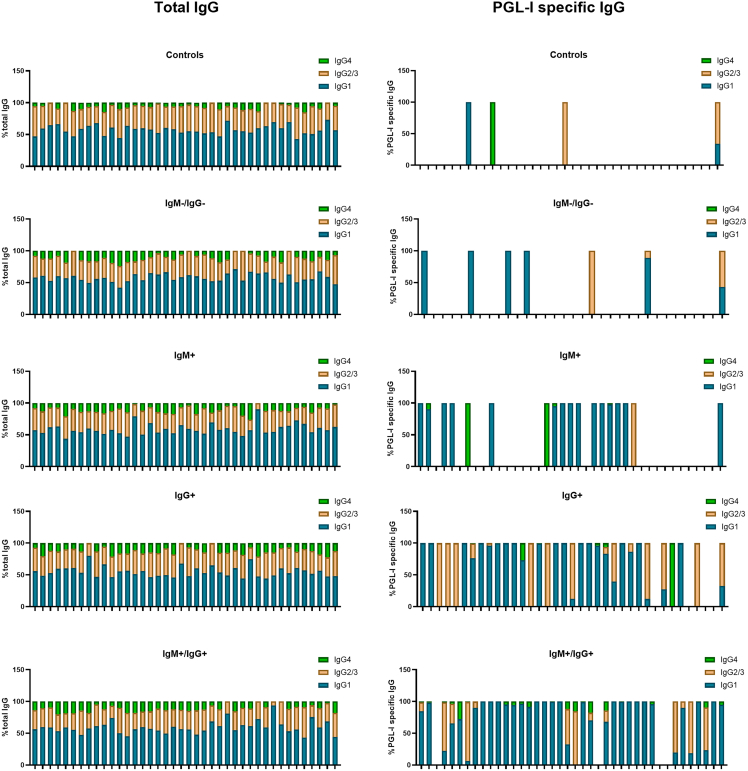
Distribution of IgG1, IgG2/3, and IgG4 in leprosy patients diagnosed in the Netherlands and controls The percentage of IgG1, IgG2/3, and IgG4 was determined by dividing the absolute intensity obtained by LC-MS of a specific subclass (i.e., IgG1) by the absolute intensity of the sum of all subclasses (IgG1 + IgG2/3 + IgG4). Each bar represents the percentage of IgG1, IgG2/3, and IgG4 of total (left column) or PGL-I-specific IgG (right column) of one individual. Color code: IgG1: blue; IgG2/3: yellow; IgG4: green.

PGL-I-specific IgG was detected in 98 individuals of the Dutch cohort and 21 individuals of the Brazilian cohort by liquid chromatography-mass spectrometry (LC-MS). As expected, the absolute intensity of PGL-I-specific IgG determined by LC-MS was highest in those individuals classified as IgG+ by ELISA (Figure S3). PGL-I-specific IgG was detected by LC-MS in 10%/7.4% of the controls, 31%/25% of patients in whom no PGL-I-specific antibodies or only IgM was detected by conventional methods and in 90/100% of patients with anti-PGL-I IgG detected by ELISA, in the Dutch and Brazilian cohort, respectively. The subclass distribution of PGL-I-specific IgG did not resemble the subclass distribution of total IgG (Figure 3). Per individual, PGL-I-specific IgG consisted generally of a single IgG subclass, with only few individuals presenting PGL-I IgG1, IgG2/3, and/or IgG4 simultaneously. IgG1 was the most frequently observed PGL-I-specific subclass (*n* = 71), but individuals with predominant IgG2/3 (*n* = 23) or IgG4 (*n* = 4) were also observed. In the Brazilian cohort, only PGL-I-specific IgG1 was detected, confirming the predominance of this IgG subclass.

### Glycosylation patterns of PGL-I-specific IgG

**Figure 4:**
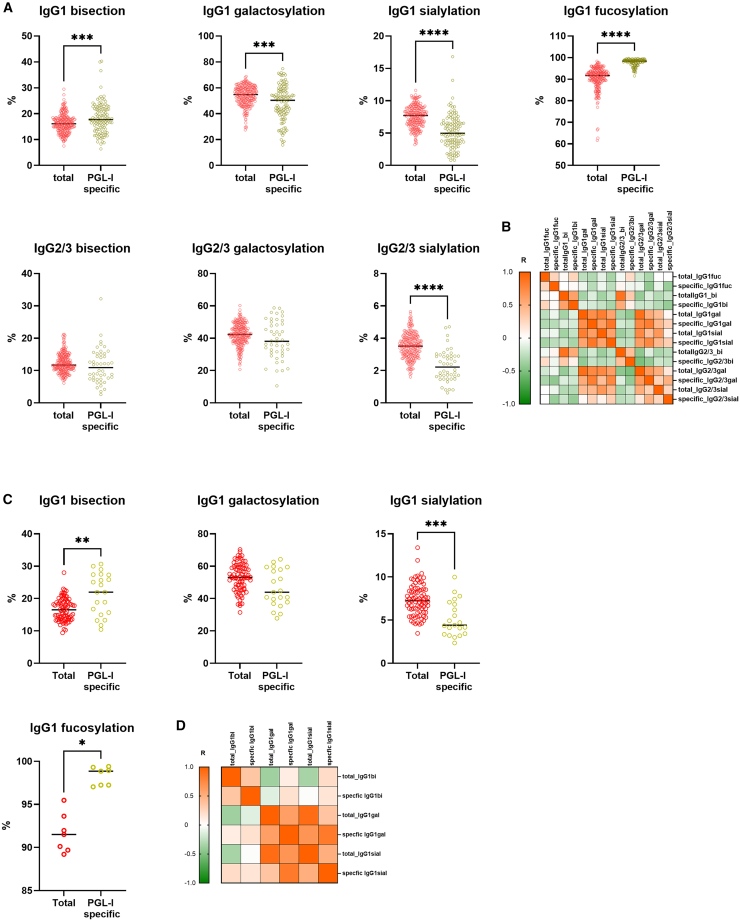
Glycosylation profile of total IgG and *M. leprae* PGL-I-specific IgG determined by LC-MS (A) Percentage of bisection, galactosylation, sialylation, and fucosylation of total (*n* = 200) and PGL-I-specific IgG1 (*n* = 114) and IgG2/3 (*n* = 46) determined in the Dutch cohort (scatterplot; line at median). A paired Wilcoxon test was performed to determine the difference between the glycosylation of total and PGL-I-specific IgG1 and IgG2/3. *p* value: ∗*p* < 0.05; ∗∗*p* < 0.01; ∗∗∗*p* < 0.001; ∗∗∗∗*p* < 0.0001. (B) Correlation matrix of glycosylation traits (percentage) of total and PGL-I-specific IgG (Pearson R). Positive correlations are indicated in orange, and negative correlations in green. The more intense the color, the higher the Pearson R value. (C) Percentage of bisection, galactosylation, and sialylation of total (*n* = 82) and PGL-I-specific IgG1 (*n* = 21) determined in the Brazilian cohort (scatterplot; line at median). PGL-I specific IgG1 fucosylation is shown for the samples which passed data curation (*n* = 7). (D) Correlation matrix of glycosylation traits of total and PGL-I-specific IgG1 (Brazilian cohort). Fucosylation was not included due to the low number of samples passing data curation for PGL-I specific IgG1.

The detection of anti-PGL-I-specific IgG by LC-MS enabled further analysis of these antibodies to determine the glycosylation patterns. First, glycosylation profiles were determined for total IgG1 and IgG2/3 (*n* = 200) and PGL-I-specific IgG1 (*n* = 114) and IgG2/3 (*n* = 46) of the Dutch cohort (Figure S4). The replicate positive control samples and sample pool of healthy controls (VisuCon) showed very high precision and repeatability for IgG1 Fc glycosylation and specific IgG capturing was highly consistent between experiments (Figures S5 and S6). The absence of PGL-I specific IgG glycopeptides in the healthy control pool demonstrates the specificity of the method (Figure S4). To determine the glycosylation traits of PGL-I-specific IgG, the level of fucosylation, galactosylation, sialylation, and bisection of these antibodies was pairwise compared to the levels of total IgG (Figure 4A). Anti-PGL-I-specific IgG1 antibodies were more highly bisected and fucosylated, but less sialylated and galactosylated than total IgG. These glycosylation traits of PGL-I specific IgG1 were replicated in the independent sample set from Brazil (Figure 4C). Even though fucosylation rates of PGL-I specific IgG1 could only be covered in part of this cohort (*n* = 7), high glycosylation of the specific antibodies was observed in all samples passing data curation. The glycosylation traits of PGL-I-specific antibodies correlated well with the glycosylation traits of the total IgG antibodies in both cohorts (Figures 4B and 4D).

**Figure 5:**
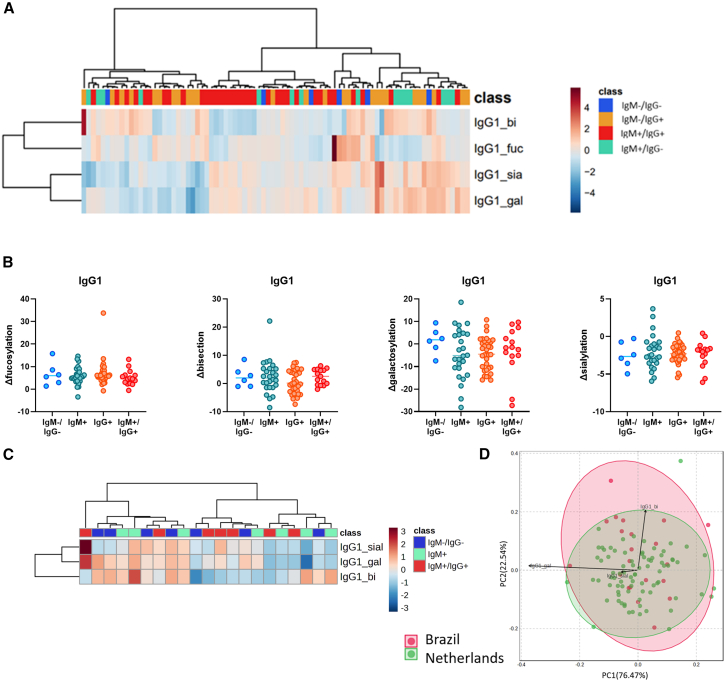
PGL-I-specific IgG1 glycosylation profiles (A) Clustered heatmap of ΔIgG1 bisection (bi), fucosylation (fuc), sialylation (sia), and galactosylation (gal) determined in the Dutch cohort. Distance measure using Euclidean, and clustering algorithm using Ward’s linkage. The dendrogram of individual patients indicates the group by color: IgM-/IgG-in blue; IgM+ in green; IgG+ in orange, and IgM+/IgG+ in red. (B) Scatterplots (line at median) indicate the ΔIgG1 bisection, fucosylation, sialylation, and galactosylation per group (Dutch cohort). Delta (Δ) values were obtained by subtracting total IgG glycosylation levels from anti-PGL-I IgG glycosylation levels. Statistical significance was determined by the Kruskal-Wallis test with Dunn’s correction for multiple testing. (C) Clustered heatmap as depicted under (A) for the Brazilian cohort. (D) PCA biplot of PGL-I-specific IgG1 glycosylation profiles, including all Dutch (green) and Brazilian (red) samples.

Glycosylation is known to be affected by age and gender, as demonstrated in the Dutch cohort (Figure S7). To account for this effect, the delta value was calculated for individuals in whom specific antibodies were detected, subtracting total IgG glycosylation levels from anti-PGL-I IgG glycosylation levels. Indeed, normalizing to total IgG eliminated the confounding effects of age and gender (Figure S7), and these delta values were used further to examine the glycosylation profile of PGL-I-specific IgG1, the predominant subclass. Glycosylation profiles of anti-PGL-I IgG1 were highly diverse and did not differ between the patient groups stratified by PGL-I antibodies (Figure 5), nor were the profiles associated with leprosy patient classification (Figure S8). PGL-I-specific IgG1 glycosylation patterns also did not discriminate between patients originating from Dutch or Brazilian outpatient clinics (Figure 5D), reflecting the similar glycoprofile observed between cohorts (Figure 4).

### Glycosylation profiles of total IgG in leprosy patients and controls

As control individuals lack PGL-I-specific antibodies, the difference in glycosylation profiles between patients and controls could only be determined for total IgG (Figures S9 and S10). Inboth cohorts, galactosylation of total IgG1 was significantly lower in leprosy patients compared to controls. Total IgG1 galactosylation might, therefore, also be associated with leprosy disease pathogenesis. Differences in total IgG bisection and sialylation were also observed between Brazilian patients and controls (Figure S10). However, the effect of age and gender on glycosylation in this cohort should be considered, as these groups were not age- and gender-matched.

### Antibody glycosylation in leprosy patients during treatment

**Figure 6:**
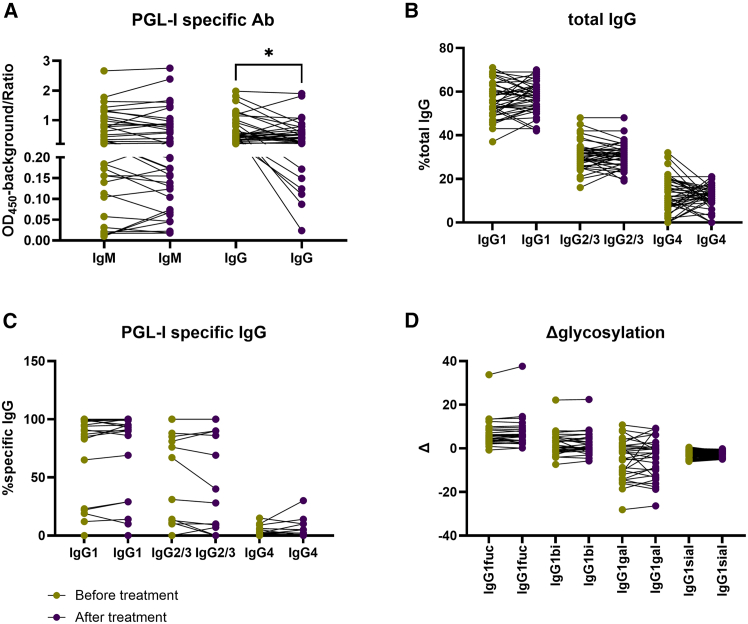
Longitudinal antibody profile of leprosy patients Two samples were collected from 40 leprosy patients at intake and approximately one year after the first visit. Statistical significance was determined using the Wilcoxon matched-pairs signed-rank test. ∗*p* < 0.05. (A) The ratio values for IgM and OD_450_ (-background) for IgG were determined by lateral flow assays or ELISA, respectively, at both time points. (B) The percentage of total IgG1, IgG2/3, and IgG4 was determined by dividing the absolute intensity obtained by LC-MS of a specific subclass (i.e., IgG1) by the absolute intensity of the sum of all subclasses (IgG1 + IgG2/3 + IgG4). (C) The percentage of PGL-I-specific IgG1, IgG2/3, and IgG4 was determined by dividing the absolute intensity obtained by LC-MS of a specific subclass (i.e., PGL-I specific-IgG1) by the absolute intensity of the sum of all subclasses (PGL-specific IgG1 + IgG2/3 + IgG4). (D) Delta (Δ) values were obtained by subtracting total IgG glycosylation levels from anti-PGL-I IgG glycosylation levels. Fuc: fucosylation, bi: bisection, gal: galactosylation, sial: sialylation.

To determine the effect of treatment on antibody characteristics in leprosy patients, a second time point was included for a subset of 40 individuals with detectable anti-PGL-I IgG by ELISA at the first time point. This sample was collected approximately 1 year after admission, during which the patient received treatment. Anti-PGL-I IgG levels detected by ELISA were significantly decreased at follow-up, whereas IgM levels were not affected in these individuals (Figure 6). This decrease was not reflected in the LC-MS data; both total and PGL-I-specific IgG1, IgG2/3, and IgG4 did not significantly change over time (Figures 6 and S11). Moreover, ΔIgG1 fucosylation, bisection, galactosylation, and sialylation also remained similar during this one-year period. In this cohort, antibody glycosylation in leprosy patients thus remained stable after one year.

## Discussion

The function of *M. leprae*-specific antibodies, which do not seem to confer protection based on their correlation with bacterial load,^13^^,^^14^ is largely unknown. To this end, antibody isotypes, subclass distribution, and glycosylation patterns were determined for total and *M. leprae*-specific antibodies in two independent cohorts, including leprosy patients from outpatient clinics in a non-endemic (the Netherlands) and endemic area (Brazil).

The most widely studied antibody response in leprosy patients is directed against the *M. leprae*-specific cell-wall component PGL-I.^9^^,^^10^^,^^11^ Consistent with previous results,^26^^,^^27^^,^^28^ PGL-I-specific IgM was the most frequently detected isotype in patients, underscoring its utility for diagnostic tools.^13^ However, the workflow for analyzing antigen-specific IgM glycosylation has not yet been established, sensitivity and precision issues still need to be addressed before anti-PGL-I IgM glycosylation analysis can be achieved. The mechanism of the predominant IgM response, which is generally produced as the first response to a pathogen, over the affinity-matured class-switched IgG and IgA, is unknown. Although less frequently detected, anti-PGL-I IgG is described as a better indicator of disease in patients than IgM.^27^ Animal studies suggest that the ability to produce anti-PGL-I IgG is associated with delayed dissemination^29^ and, in combination with low anti-PGL-I IgM, less severe disease^30^ or protection against disease after vaccination.^31^ In the human study described herein, patients presenting solely with anti-PGL-I IgG were rare (3%), and IgG was primarily detected in patients with high bacterial loads (LL/BL). The same pattern was observed for IgA,^28^^,^^32^^,^^33^ indicating that all PGL-I-specific antibody isotypes assessed were most frequently generated in patients who present with disseminated infection.

Further characterization of anti-PGL-I IgG demonstrated that these antibodies were mainly of the IgG1 subclass, requiring T cell help for their production. Of the four IgG subclasses, IgG1 is one of the most potent mediators of antibody effector functions via complement or Fcγ receptors.^34^ The Fc domain of PGL-I-specific IgG1 was more highly bisected and fucosylated, but less sialylated and galactosylated compared to total IgG1. High fucosylation is known to reduce antibody binding to FcγRIIIA and FcγRIIIB on innate immune cells. As a result, fewer NK cells, monocytes, macrophages, and granulocytes are triggered to kill infected cells.^23^^,^^35^ Fewer NK cells were indeed observed in the circulation of leprosy patients with high bacterial loads,^36^ suggesting a less effective innate response in these individuals with high anti-PGL-I antibody levels. Reduced galactosylation and sialylation of IgG1 decrease antibody binding to C1q, affecting further complement deposition.^19^ The observed glycosylation traits thus point to a reduced capacity of PGL-I-specific IgG1 to elicit an effective innate immune response and subsequent bacterial clearance, but functional assays are required to demonstrate that these antibodies indeed fail to activate effector mechanisms.

Glycosylation profiles were highly heterogeneous across patients and were not associated with specific disease presentations, whereas the presence of anti-PGL-I antibodies per se was clearly associated with disease presentation. Modulation of antigen-specific Fc glycosylation in infectious diseases has been reported for, among others, COVID-19,^23^^,^^37^ cytomegalovirus (CMV), human immunodeficiency virus-1 (HIV-1), hepatitis B virus (HBV),^23^ dengue,^38^^,^^39^ and tuberculosis.^16^^,^^24^^,^^40^ In contrast to the high fucosylation observed for anti-PGL-I IgG1, antigen-specific IgG1 antibodies in many other infectious diseases show varying levels of afucosylation.^23^^,^^24^^,^^35^^,^^41^ In these studies, the elevated binding of afucosylated antibodies to FcγRIII induced either protective responses or triggered excessive inflammation. Whether the high fucosylation of anti-PGL-I IgG1 oppositely reduces the binding to FcγRIII requires further study.

PGL-I-specific IgG1 glycoprofiles in leprosy patients assessed longitudinally remained stable during the one-year time frame. This is in contrast to the dynamic changes observed in IgG1 glycosylation patterns in COVID-19, reversing from a pro-to anti-inflammatory state from hospitalization to discharge,^23^ and a decrease in inflammatory Fc glycans during effective tuberculosis treatment, implicating a correlation with disease activity and the presence of replicating bacteria in these individuals.^40^ Treatment efficacy among patients with leprosy assessed longitudinally was unavailable, impeding stratification by treatment success. During effective treatment, anti-PGL-I antibodies generally decrease,^42^^,^^43^^,^^44^ providing an indication of the treatment effect. The determined anti-PGL-I IgM and IgG levels did not decrease after one year in 40% and 53% of patients, respectively, suggesting a heterogeneous treatment response in this cohort.

In addition to PGL-I-specific IgG, glycoprofiles of total IgG were also assessed, showing reduced galactosylation of IgG1 in leprosy patients compared with controls. Age and gender influence the galactosylation status of these antibodies,^23^^,^^45^ which was not completely accounted for by the delta correction method performed. However, this difference persisted when matching individuals by age and gender (Dutch cohort). Low IgG galactosylation levels are observed in inflammatory diseases like multiple sclerosis, and rheumatoid arthritis, as well as in infections like tuberculosis and leishmaniasis.^46^ Moreover, total IgG1 galactosylation has been described as a potential biomarker of immune activation, inversely correlating with inflammatory marker CRP and parasitic infections that dampen the immune response.^46^ The reduced galactosylation levels observed in leprosy patients compared to healthy controls may thus be indicative of the ongoing *M. leprae* infection.

Total IgG isotypes also showed consistent differences between patients and controls across cohorts, with lower IgG2/3 and higher IgG4 levels in leprosy patients. IgG3 is the most potent complement activator and inducer of phagocytosis of the four subclasses, binding with high affinity to Fc receptors, while IgG4 is the least potent in inducing these effector functions.^47^ IgG3 deficiency is associated with a history of recurrent infections leading to chronic lung disease.^48^ The reduced total IgG3 in leprosy patients suggests that the lower levels of this potent antibody subtype impede their ability to clear *M. leprae* efficiently. To induce IgG4, prolonged or repeated antigen exposure is required. IgG4 is regarded as an anti-inflammatory antibody, but is also described to have detrimental roles in autoimmunity.^49^ In individuals infected with another mycobacterium, *M. tuberculosis*, the IgG4 response to *M. tuberculosis* antigens correlated with active disease.^40^ In this study, IgG4 was hardly detected in response to PGL-I, indicating that other *M. leprae*-specific antigens may contribute to the IgG4 response in leprosy patients. However, IgG4 was undetectable or low in response to other frequently studied *M. leprae* antigens in the context of leprosy diagnosis i.e., LID-1 (fusion protein product of ML0405 and ML2331) and lipoarabinomman (LAM; cell wall component of *M. leprae*).^50^ In this respect it is interesting that a pathological role of host antigen-specific IgG4 antibodies in nerve demyelination has been described in chronic inflammatory demyelinating polyneuropathy, an acquired autoimmune disorder affecting the peripheral nervous system.^51^^,^^52^^,^^53^ The elevated IgG4 levels observed in leprosy patients in this study could thus point toward a similar mechanism for *M. leprae*-induced nerve damage. Potential neural antigens for this IgG4 response are myelin protein zero, ceramide and myelin basic protein (MBP), to which antigen-specific IgM,^54^ and total Ig^55^/IgG,^56^ respectively, were significantly elevated in leprosy patients across the spectrum. Anti-MBP antibodies correlated with the number of nerves involved in leprosy patients, and molecular mimicry between this host antigen and *M. leprae* proteins 50S ribosomal L2 and lysyl tRNA synthetase has been described,^56^ implying a role for these neural antibodies in the pathogenesis of leprosy.

In summary, this study reproducibly determined the glycosylation profile of PGL-I-specific IgG and described glycoprofiles in leprosy patients. The glycoprofile of the predominant IgG subtype observed, IgG1, is generally linked to reduced effector functions. The exact function of anti-PGL-I IgG1 remains to be elucidated, though the association of the identified glycoprofile with dampened effector functions is in line with their abundance in individuals with high bacterial loads. Unraveling the function of *M. leprae*-specific antibodies further will provide insight into the disease mechanism and could identify new targets for diagnostic tests^57^ and treatment.

### Limitations of the study

The most frequently detected antibody isotype in response to PGL-I was IgM. Glycosylation of this isotype could not be determined, as analyzing antigen-specific IgM glycosylation has not yet been established. Notably, the antigen-specific IgM glycosylation is poorly understood, potential differences in anti-PGL-I IgM glycosylation will be hard to interpret, as we do not know how glycosylation influences IgM function. Due to the unavailability of data on treatment efficacy for the patients longitudinally assessed, interpretation of the stable glycoprofiles observed is also limited. To determine whether these stable profiles are related to either biological irreversibility or poor treatment response, requires evaluation in prospective studies. Additional research is also required to determine whether the glycosylation patterns observed indeed result in diminished effector functions of PGL-I specific IgG.

## Resource availability

### Lead contact

Requests for further information should be directed to the lead contact Anouk van Hooij (a.van_hooij@lumc.nl).

### Materials availability

This study did not generate new unique reagents.

### Data and code availability

• All data supporting the findings of this study are available within the main manuscript and the supplementary files provided
• This paper does not report original code
• Any additional information required to reanalyze the data reported in this paper is available from the lead contact upon request.

## Acknowledgments

The authors thank all patients and contacts for their voluntary participation and Sanne Geboers, Els Hartsema, Lotte Sikkema, Els Verhard, and Zijie Zhou for their lab assistance with screening patient serum for anti-PGL-I antibodies. Research reported in this study was supported by The Leprosy Mission Great Britain (TLMGB) and the Q.M. Gastmann-Wichers foundation (A.G.).

## Author contributions

Conceptualization: A.v.H., and A.G.; human sample and data collection: C.L.M.v.H, C.S.d.M., R.O.M., and A.G.; sample preparation and experimental work: A.v.H. and C.S.d.M.; LC-MS experiments and data collection: W.W., S.G., and J.N.; data analysis and writing manuscript draft: A.v.H. and W.W.; manuscript review: A.G., C.S.d.M., and M.W.; assistance data analyses and interpretation: A.G. and M.W.; funding acquisition: A.G. All authors read and approved the final version of the manuscript.

## Declaration of interests

The authors declare no competing interests.

## STAR★Methods

### Key resources table

**Table:** 

REAGENT or RESOURCE	SOURCE	IDENTIFIER
**Antibodies**		

RaG	Sigma-Aldrich	G4018; RRID: AB_259895
Anti-IgM	Sigma-Aldrich	I0759; RRID: AB_260109
anti-IgG-HRP	Sigma-Aldrich	A0170; RRID: AB_257868
anti-IgA-HRP	Sigma-Aldrich	I0884; RRID: AB_260111

**Chemicals, peptides, and recombinant proteins**		

NaCl	Merck	#1.06404.500
BSA	Sigma	# A2153
Triton X-100	Merck	X100
Na_2_CO_3_	Merck	1.06392.1000
NaHCO_3_	Merck	1.06329.1000
H_2_SO_4_	Merck	1.00731.1010

**Software and algorithms**		

LaCyTools		
Graphpad Prism	Dotmatics	V10.2.3
R Studio	Posit PBC	v2023.12.1

**Other**		

ND-*O*-HSA	BEI Resources	NR-59499
Flat-Bottom Immuno Nonsterile 96-Well PolySorp NUNC plates	Thermo Fisher Scientific	735–0131
PBS	Sigma-Aldrich	D8537
Tween 20	Merck	# 8221840500
Spectramax i3x microplate reader	Molecular Devices	
Impact quadrupole time-of-flight mass spectrometer	Bruker	
3,3′,5,5′-tetramethylbenzidine	Thermo Fisher Scientific	10647894
200 nm, NaYF4:Yb3+,Er 3+) functionalized with polyacrylic acid	Intelligent Material Solutions Inc.	
10 mm glass fiber sample/conjugate pad	Kenosha	Ahlstrom
25 mm laminated nitrocellulose membrane	Kenosha	Sartorius UniSart CN95
20 mm absorbent pad	Kenosha	Whatman Cellulose 470

### Experimental model and study participant details

#### Biobank Dutch outpatient clinic

The LUMC biobank consists of sera from leprosy patients diagnosed in the Netherlands since 1981. 831 unique patients were included in this study, with varying numbers of follow-up timepoints available after intake. Leprosy is not endemic in the Netherlands and most cases were foreign born. Age and gender information, as well as leprosy classification according to the Ridley-Jopling classification system^6^ were available (Table S1). This five-type classification system is based on histological and immunological features, ranging from the self-limiting tuberculoid (TT) to disseminated lepromatous leprosy (LL). In between these two poles of the spectrum, patients are classified as borderline tuberculoid (BT), mid-borderline (BB) or borderline lepromatous (BL) patients. Ethnic information was not available.

#### Dutch healthy controls

Healthy controls selected for the glycosylation analysis (*n* = 40) were age and gender-matched to the patient groups stratified by PGL-I antibody response (40 per group) when possible (Figure S1). These controls were Dutch inhabitants of Western-European descent (*n* = 22) or descent from leprosy endemic areas (*n* = 18) to mimic the genetic diversity of the patient cohort. The effect of age and gender on the study results was evaluated (Figures 2 and S7).

#### Brazilian outpatient clinic

Patients were recruited at the Souza Araujo Outpatient Clinic (Oswaldo Cruz Foundation-FIOCRUZ, Rio de Janeiro-RJ), and serum samples were kept at the Biorepository of the Leprosy Laboratory (Oswaldo Cruz Institute, FIOCRUZ, Rio de Janeiro-RJ). Research participants signed a consent form. Fifty-five patients diagnosed according to the Ridley-Jopling classification system were recruited (17 LL, 12 BL and 26 BT). Age and gender information was available (Table S3), the ethnicity of participants was unknown. The exclusion criteria for the study were: individuals over 65 years of age or under 18 years of age, individuals known to be HIV positive, individuals with chronic or acute diseases unrelated to leprosy, individuals infected with any infectious agent, and individuals with chronic inflammatory diseases, who were taking immunosuppressive medications, or who were pregnant.

#### Brazilian healthy controls

The Brazilian healthy control group consists of individuals (*n* = 27) without a diagnosis of leprosy, aged 18 to 65 years (Table S3). The exclusion criteria for the group were: being in- or peridomicillary contact with patients with leprosy, being over 65 years of age or under 18 years of age and having any acute or chronic disease of any etiology known to be diagnosed at the time of blood collection. The controls samples were not age- and gender matched to the patient group, this should be considered when interpreting the findings of the Brazilian cohort.

#### Ethical approval

Ethical permission was received from a local ethical board in the Netherlands (MEC-2012-589) and from Fiocruz Research Ethics Committee (approval number 7.004.067, CAAE 79475324.8.0000.5248).

### Method details

#### Anti-PGL-I IgM lateral flow assay

Lateral flow (LF) strips detecting PGL-I-specific IgM were produced as described previously.^58^^,^^59^ Serum samples were diluted 50-fold in assay buffer (100 mM Tris pH 8, 270 mM NaCl, 1% (w/v) BSA, 1% (v/v) Triton X-100) prior to application to the anti-PGL-IgM LF strips. 50 μL of diluted serum was applied to the LF strips and immunochromatography was allowed to continue for at least 30 min until dry. LF strips were analyzed using a dedicated benchtop reader (UPCON; Labrox, Finland). Results are displayed as the ratio value between Test and Flow-Control signal based on relative fluorescence units (RFUs; excitation at 980 nm and emission at 550 nm) measured at the respective lines.

#### Anti-PGL-I antibody ELISA

ELISAs were performed to determine the presence of IgG or IgA antibodies against ND-*O*-HSA (PGL-I-Based Glycoconjugate of Human Serum Albumin), which consists of the synthetic immunodominant natural disaccharide (ND) part of the *M. leprae* phenolic glycolipid antigen coupled to human serum albumin (HSA). Synthetic PGL-I was obtained through BEI Resources, NIAID, NIH: *Mycobacterium leprae*, ND-*O*-HSA (PGL-I-Based Glycoconjugate of Human Serum Albumin), NR-59499 (https://www.beiresources.org/Catalog/antigen/NR-59499.aspx).

Clear Flat-Bottom Immuno Nonsterile 96-Well PolySorp NUNC plates (Thermo Fisher Scientific) were coated with 200 ng synthetic PGL-I (Product#:19347 www.beiresources.org) in 0.1 M Na_2_CO_3_/NaHCO_3_ coating buffer, pH 9.6. 0.1% BSA in coating buffer was used as background control. Coated plates were incubated overnight at 4°C and subsequently washed three times with 200 μL PBS/0.05%Tween 20. Per well, 200 μL PBS/1%BSA/0,05% Tween 80 was added and incubated at room temperature for at least 1 h. After incubation, the blocking buffer was removed and 50 μL of 1:400 diluted plasma was added to a well coated with synthetic PGL-I and 50 μL to a well containing 0.1% BSA for each sample. After 2 h of incubation at room temperature, the wells were washed three times as described above. Per well, 50 μL anti-IgG-HRP (1:4000; Sigma A0170) or anti-IgA-HRP(1:8000; Sigma I0884) in PBS/0.05% Tween 20 was added and incubated at room temperature for 2 h. After washing the ELISA plate four times with the procedure described above, 50 μL 3,3′,5,5′-tetramethylbenzidine (TMB, Thermo Fisher Scientific, Bleiswijk, the Netherlands) was added allowing the color reaction to start. The reaction was stopped after 10 min by adding 50 μL 1M H_2_SO_4_. Absorbance was determined at a wavelength of 450 nm (Spectramax i3x microplate reader).

#### IgG Fc glycosylation analysis

We applied the GLYcoLISA workflow for analyzing the Fc glycosylation of total IgG and antigen-specific IgG,^60^ with a few adjustments as follows. Total IgG was enriched from 1 μL serum using protein G, whereas antigen-specific IgG was affinity-captured using the synthetic PGL-I coated Clear Flat-Bottom Immuno Nonsterile 96-Well PolySorp plates (Thermo Fisher Scientific). Briefly, 96-well Polysorp plates were coated with 180 μL of synthetic PGL-I at a concentration of 4 μg/mL in PBS at 4°C overnight. After washing with 0.05% Tween 20 in PBS, PBS/1% BSA/0.05% Tween 80 as a blocking buffer was added and incubated for 1 h at room temperature. Subsequently, 10-fold diluted serum was added to the coated plates and incubated for 2 h at room temperature while shaking. Plates were washed, and adsorbed IgGs were eluted using 150 μL 100 mM formic acid spiked with 2 ng of a stable isotope-labeled IgG standard for absolute quantitation,^61^ vacuum dried, and cleaved with trypsin to generate IgG Fc glycopeptides. IgG Fc glycopeptides were registered by nano-liquid chromatography-mass spectrometry (LC-MS) using an Impact quadrupole time-of-flight mass spectrometer (Bruker Daltonics, Bremen, Germany). IgG Fc glycopeptides were assigned based on accurate mass and retention time, and relatively quantified using LaCyTools.^60^ A plasma frozen pool from a minimum of 20 healthy controls (VisuConF, Affinity Biologicals, Ancaster, Canada) and a positive serum sample were used as quality control for the assay.

### Quantification and statistical analysis

To stratify groups based on anti-PGL-I antibody levels, samples with a Ratio value > 0.2 or an OD_450_-value corrected for background >0.2 were considered seropositive for anti-PGL-I IgM or IgG, respectively. This stratification results in four groups based on PGL-I antibody level: seronegative (IgM-/IgG-), IgM positive only (IgM+), IgG positive only (IgG+) or double-positive (IgM+/IgG+). To analyze the Brazilian cohort, the IgM+, IgG+ and IgM+/IgG+ were combined as one seronegative group due to the individual group sizes.

Groups were cross-sectionally compared using Mann-Whitney U tests (two groups) or Kruskal-Wallis with Dunn’s correction for multiple testing (multiple groups). The Wilcoxon matched pairs signed-rank test was used to determine the differences between two paired groups. The cross-sectional analysis, Pearson correlation, and linear regression analysis were performed using Graphpad Prism (V10.2.3). P-values <0.05 were considered statistically significant (∗*p* < 0.05; ∗∗*p* < 0.01; ∗∗∗*p* < 0.001; ∗∗∗∗*p* < 0.0001). Clustered heatmaps and principal component analysis (PCA) biplots were computed using R Studio (v2023.12.1).

The IgG subclass distribution was determined by dividing the absolute intensity obtained by LC-MS of a specific subclass (i.e., IgG1) by the absolute intensity of the sum of all subclasses (IgG1 + IgG2/3 + IgG4). Delta (Δ) values were obtained by subtracting total IgG glycosylation levels from anti-PGL-I IgG glycosylation levels.

## Supplemental information

Document S1. Figures S1–S11 and Tables S1 and S3

Table S2. Age, gender and anti-PGL-I antibody status of samples selected for IgG Fc glycosylation analysis by LC-MS
