# Supplementary material for: Glycosylation profile of Mycobacterium leprae-specific antibodies associated with disseminated infection
Source: iScience. 2026 Jun 20;29(7):116373. doi: 10.1016/j.isci.2026.116373 (PMC13315973; doi:10.1016/j.isci.2026.116373)
Supplement: Document S1. Figures S1–S11 and Tables S1 and S3 [file mmc1.pdf]

iScience, Volume *29*

Supplemental information

Glycosylation profile of *Mycobacterium leprae*-specific antibodies associated with disseminated infection

Anouk van Hooij, Wenjun Wang, Cristiana Santos de Macedo, Jan Nouta, Steinar Gijze, Roberta Olmo Pinheiro, Colette L.M. van Hees, Manfred Wuhrer, and Annemieke Geluk

Supplemental Figures

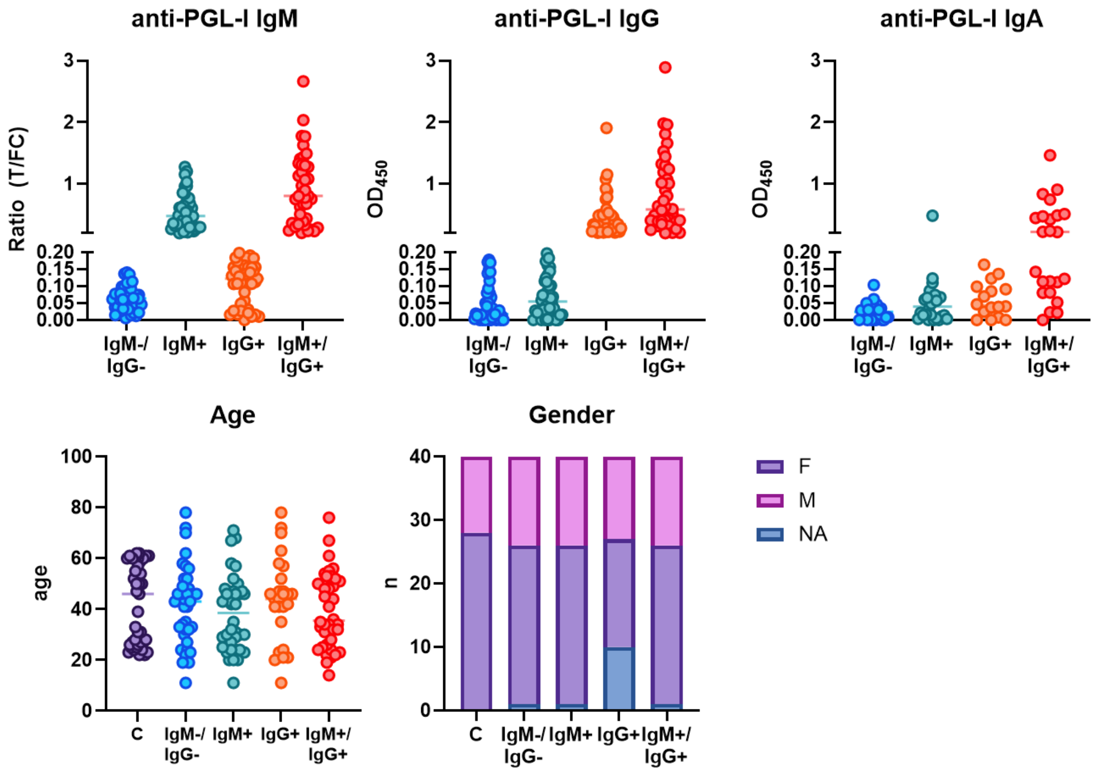

Supplementary Figure S1: Characteristics of the sample selection based on PGL-I isotype. Leprosy patients were selected based on anti-PGL-I antibody status, resulting in four groups of 40 individuals: seronegative (IgM-/IgG-), IgM-positive only (IgM+), IgG-positive only (IgG+), and double-positive (IgM+/IgG+). To stratify groups by anti-PGL-I antibody levels, samples with a Ratio>0.2 (*y*-axis) or an OD450 value corrected for background > 0.2 (*y*-axis) were considered seropositive for anti-PGL-I IgM or IgG, respectively. PGL-I IgA was determined by ELISA for these groups as well. Forty healthy Dutch individuals were included as the control group (C). These groups did not significantly differ in age or gender distribution. Scatter plots with line at median. F: female; M: male; NA: not available.

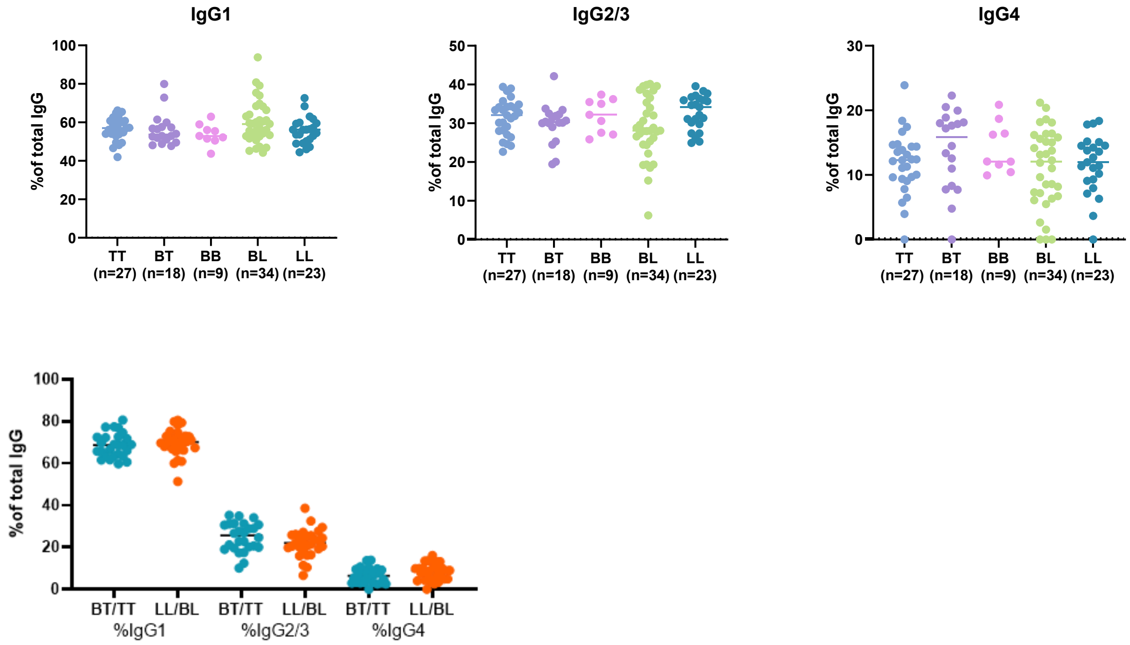

Supplementary Figure S2: Total IgG1, IgG2/3, and IgG4 distribution in leprosy patients stratified by classification. The percentage of IgG1, IgG2/3, and IgG4 was determined by dividing the absolute intensity obtained by LC-MS of a specific subclass (i.e., IgG1) by the absolute intensity of the sum of all subclasses (IgG1 + IgG2/3 + IgG4). The %IgG subclasses were compared between groups by Kruskal-Wallis with Dunn's correction for multiple testing. Results are shown for patients stratified by classification; Scatter plots with line at median. Dutch cohort (top panels) lepromatous (LL; n=23), borderline lepromatous (BL; n=34), midborderline (BB; n=9), borderline tuberculoid (BT; n=18), and tuberculoid leprosy (TT; n=27). Brazilian cohort (bottom panel): 29 LL/BL and 26 BT patients.

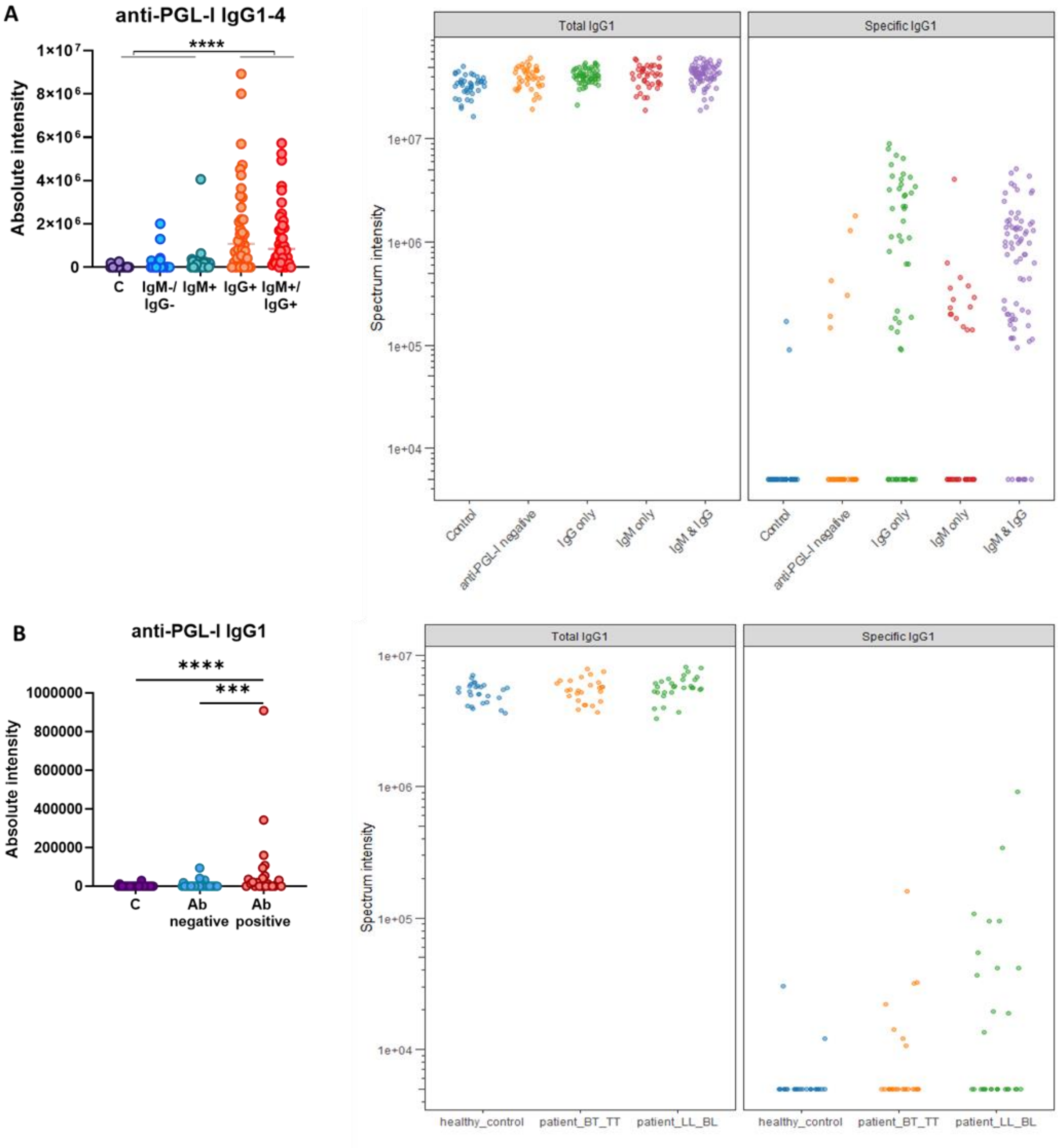

Supplementary Figure S3: **Absolute intensity of anti-PGL-I specific antibodies.** The sum of PGL-I specific IgG1, IgG2/3, and IgG4 was determined by LC-MS (absolute intensity) and compared between groups by Kruskal-Wallis with Dunn's correction for multiple testing. * $p<0.05$; *** $p<0.001$; **** $p<0.0001$. Scatter plots with line at median. (A) Dutch cohort. Groups (*x*-axis): control group (C), seronegative (IgM-/IgG-), IgM positive only (IgM+), IgG positive only (IgG+), or double positive (IgM+/IgG-). Forty individuals were included per group. (B) Brazilian cohort. Groups (*x*-axis): control group (C, n=27), seronegative (Ab negative, n=31), seropositive (Ab positive, n=27). IgG1 detected only. For both cohorts the absolute intensity (spectrum intensity) of the pooled glycopeptide signals of total and specific IgG1 were plotted, samples that failed calibration were assigned an arbitrary spectrum intensity of 8,000.

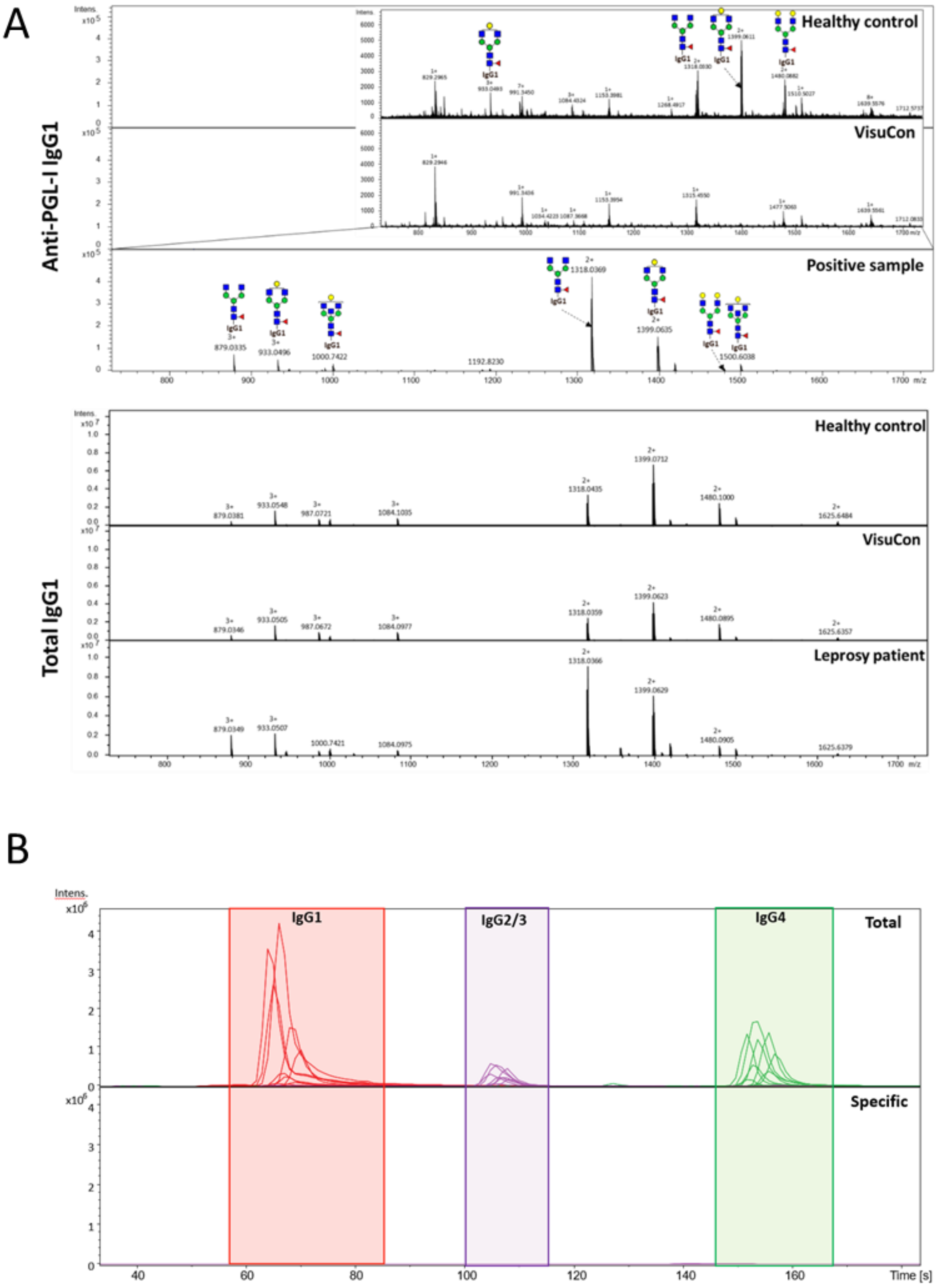

Supplementary Figure S4: Sum spectra of anti-PGL-I and total IgG1 Fc glycopeptides. (A) Sum spectra of the most abundant PGL-I-specific and total IgG1 Fc glycopeptides determined by LC-MS. Spectra are shown for one healthy control, VisuCon (plasma frozen pool of healthy controls), and one leprosy patient from the Dutch cohort. In healthy controls, few analytes were detected, whereas leprosy patients showed higher IgG1 glycopeptide intensities. (B) Extracted Ion Chromatogram of IgG subclasses from a healthy control pool (VisuCon) for total and PGL-I specific IgG glycopeptides, indicating the specificity of the method, as no specific IgG was captured in this negative control sample.

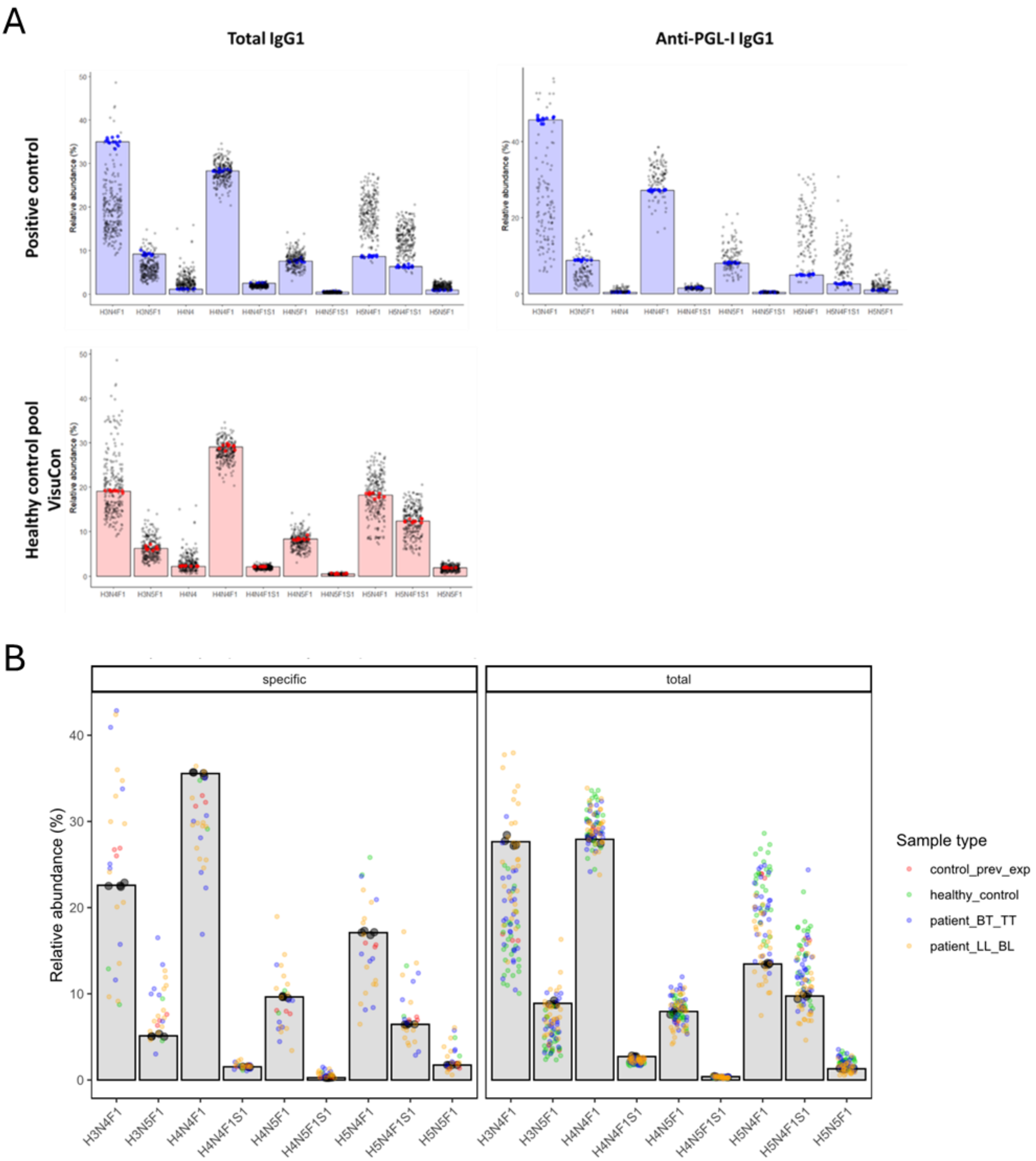

Supplementary Figure S5: Quality control IgG-FC-glycosylation analysis. (A) Replicates of a positive control sample (n=13; top panels) and a healthy control pool (Visucon; n=9; bottom panel) were included in the assay to assess the precision and repeatability of the determined IgG1 Fc. The anti-PGL-I and total IgG1 glycosylation profiles of the Dutch cohort (black dots) were plotted with replicates of the positive control (blue dots) and Visucon sample (red dots). The bars represent the mean of the replicates. (B) The anti-PGL-I and total IgG1 glycosylation profiles of the Brazilian cohort (dots coloured by sample type according to legend) were plotted with replicates of a pooled positive sample (black dots; n=4) and replicates of a control sample included in the Dutch cohort (pink; n=3). The bars represent the mean of the replicates.

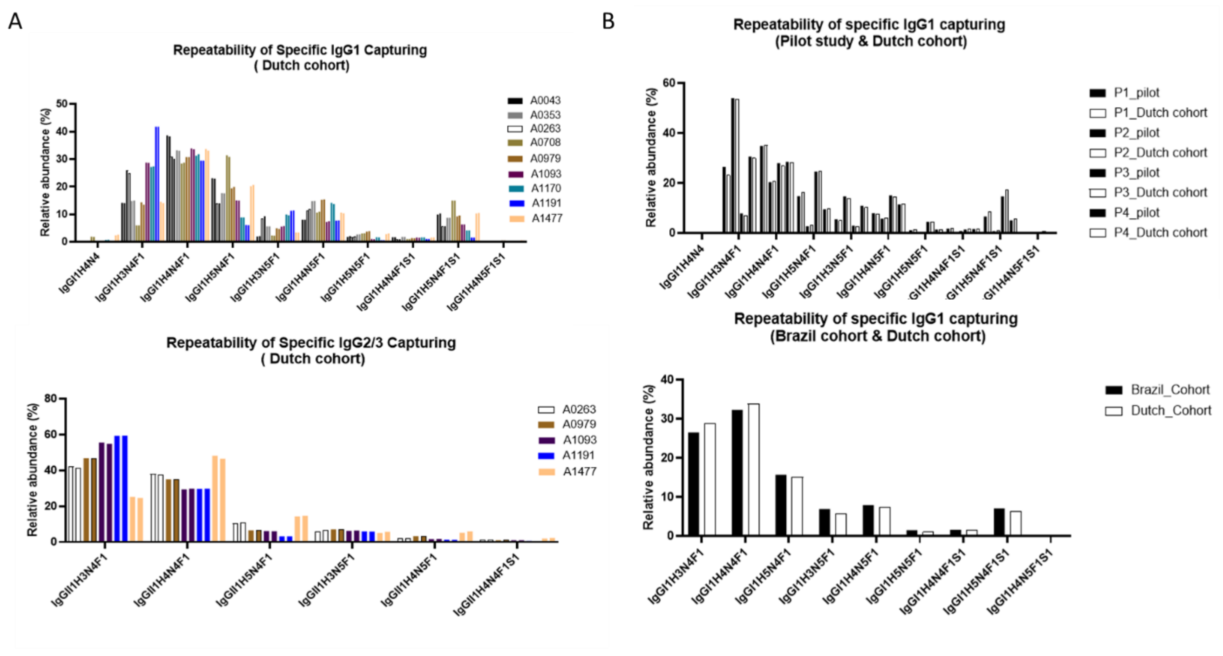

Supplementary Figure S6: Repeatability of PGL-I specific IgG capturing (A) Anti-PGL-I IgG1 and IgG 2/3 glycosylation profiles of duplicate samples included as experimental controls in the Dutch cohort, indicating very high precision and repeatability for specific IgG1 and IgG2/3 capturing intra-experimentally. (B) Evaluation of interexperimental repeatability, comparing results from the same samples assessed on different dates (black bar: day 1; white bar: day 2)

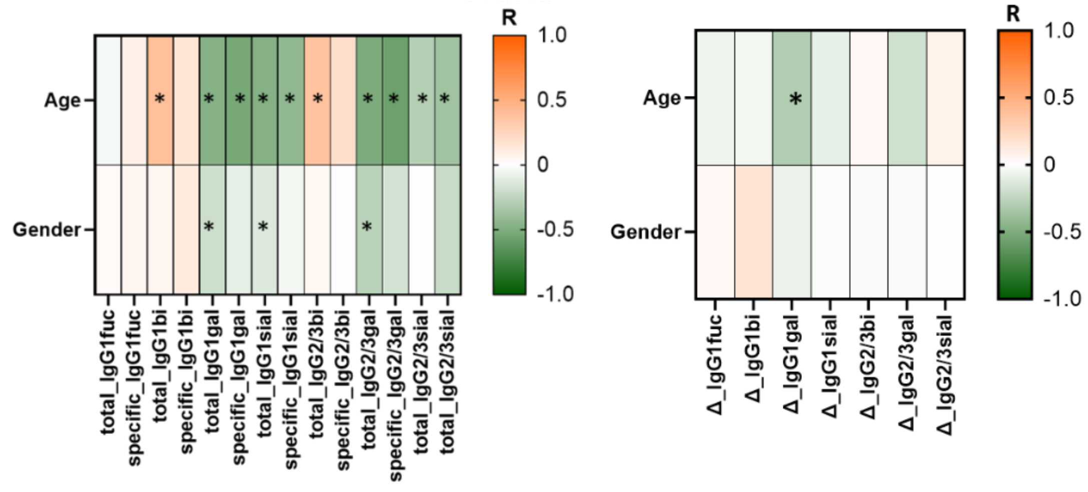

Supplementary Figure S7: Age and gender confounding effect on glycosylation. Heatmaps indicate the Pearson R value; positive correlation is displayed in orange and negative correlation in green. The more intense the colour, the higher the Pearson R value. Left panel: Correlation with possible confounders, age and gender, was determined for the percentage of total and PGL-I specific IgG1-3 fucosylation (fuc), bisection (bi), galactosylation (gal), and sialylation (sial). Significant correlation with age (10/14) and gender (3/14) were observed. Right panel: Correction for the age and gender confounding effect was performed by subtracting total IgG glycosylation levels from anti-PGL-I IgG glycosylation levels, resulting in a delta (Δ) value. * correlations with a significant *p*-value.

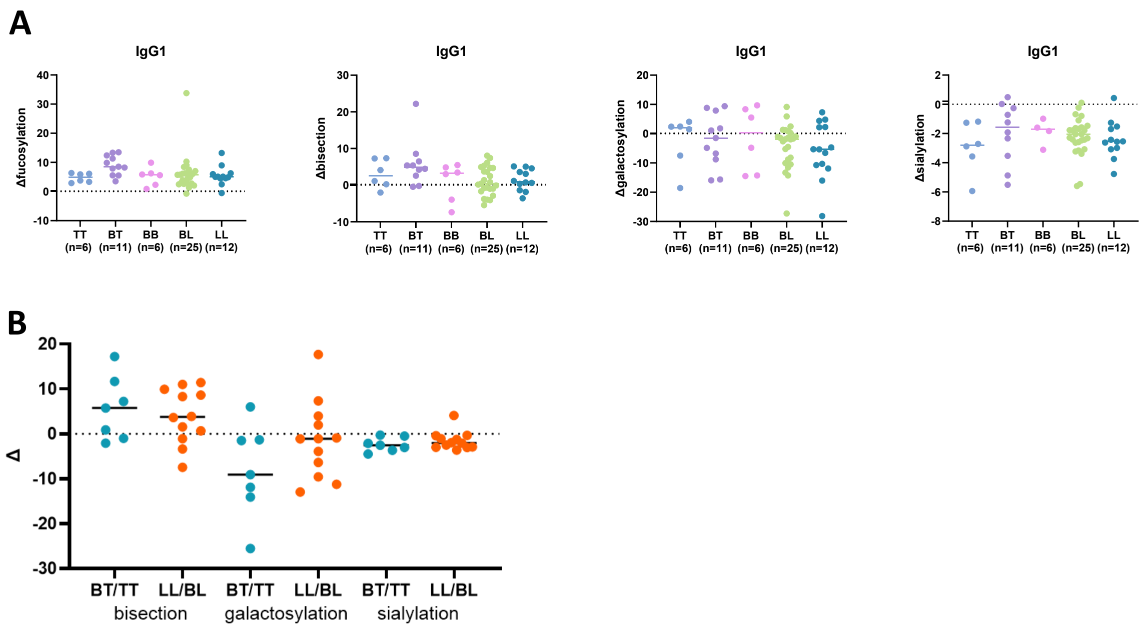

Supplementary Figure S8: **PGL-I specific glycosylation profiles in leprosy patients stratified by classification.** Scatter plots (line at median) indicate the ΔIgG1 bisection, fucosylation, sialylation, and galactosylation per group of the Dutch (A) and Brazilian (B) cohort. Delta (Δ) values were obtained by subtracting total IgG glycosylation levels from anti-PGL-I IgG glycosylation levels. Statistical significance was determined by the Kruskal-Wallis test with Dunn's correction for multiple testing. Results are shown for patients stratified by classification. Dutch cohort (top panels) lepromatous (LL; n=23), borderline lepromatous (BL; n=34), midborderline (BB; n=9), borderline tuberculoid (BT; n=18), and tuberculoid leprosy (TT; n=27). Brazilian cohort (bottom panel): 29 LL/BL and 26 BT patients.

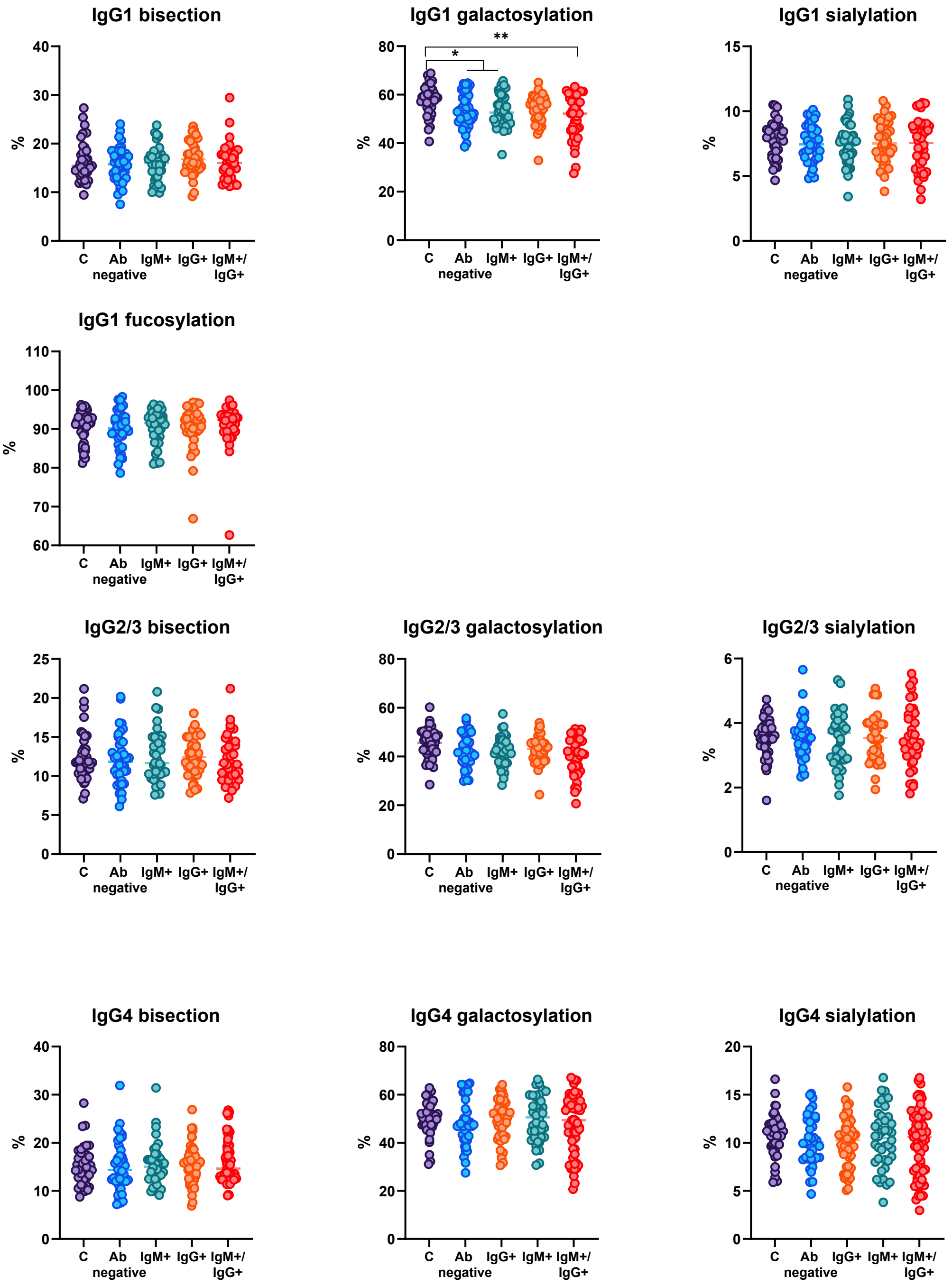

Supplementary Figure S9: Glycosylation profile of total IgG1, IgG2/3, and IgG4, Dutch cohort. Percentage of bisection, galactosylation, sialylation, and fucosylation of total IgG1, IgG2/3, and IgG4 stratified by group (Scatter plots, line at median). Groups (*x*-axis): control group (C), seronegative (IgM-/IgG-), IgM positive only (IgM+), IgG positive only (IgG+), or double positive (IgM+/IgG-). Forty individuals were included per group. Statistical differences between groups were determined by the Kruskal-Wallis test with Dunn's correction for multiple testing. *p<0.05; **p<0.01.

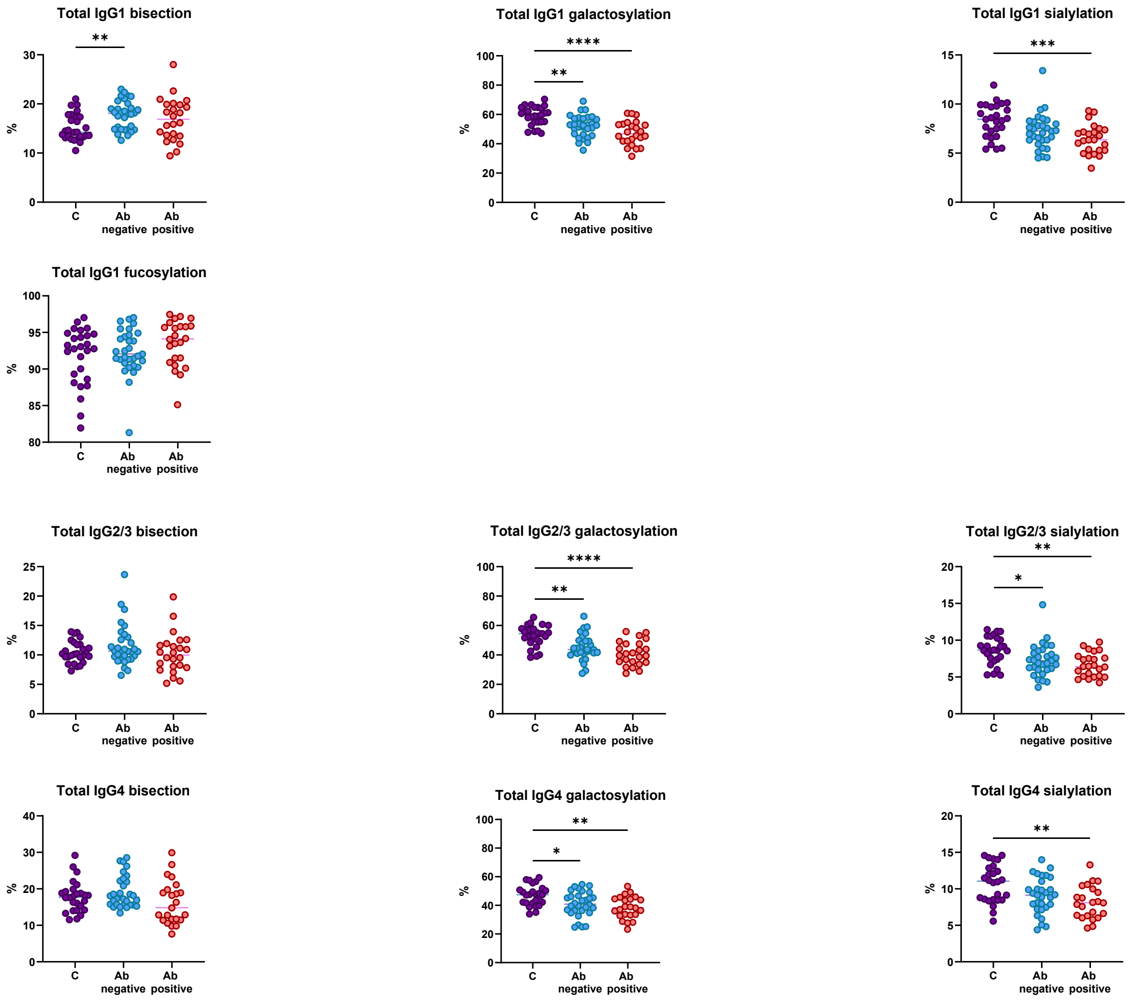

Supplementary Figure S10: Glycosylation profile of total IgG1, IgG2/3, and IgG4, Brazilian cohort. Percentage of bisection, galactosylation, sialylation, and fucosylation of total IgG1, IgG2/3, and IgG4 stratified by group (scatter plots, line at median). Groups (*x*-axis): control group (C, n=27), seronegative (Ab negative, n=31), seropositive (Ab positive, n=27). Statistical differences between groups were determined by the Kruskal-Wallis test with Dunn's correction for multiple testing. *p<0.05; **p<0.01, *** p<0.001; **** p<0.0001.

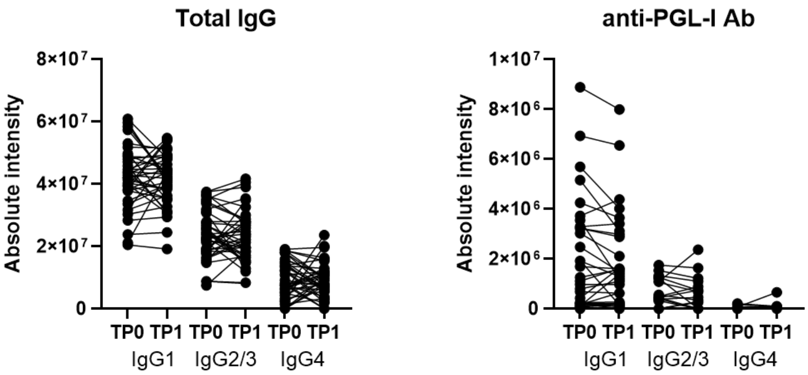

Supplementary Figure S11: Longitudinal pattern of total and anti-PGL-I specific IgG subclasses. The sum of total and PGL-I specific IgG1, IgG2/3, and IgG4 was determined by LC-MS (absolute intensity). Two samples were collected from 40 leprosy patients at intake and approximately one year after the first visit. Statistical significance was determined by the Wilcoxon matched-pairs signed rank test. No significant differences were identified.

Supplemental Tables

Supplementary Table S1. Leprosy classification, age, and gender distribution of patients collected at the Dutch outpatient clinic (n=831). LL = lepromatous leprosy; BL = borderline lepromatous; BB = midborderline; BT = borderline tuberculoid; TT = tuberculoid leprosy; M = male; F = female; NA = not available.

	**Median age (range)**	**M (%)**	**F (%)**	**NA (%)**
**All patients (n=831)**	39 (5-85)	408 (49%)	298 (36%)	129 (15%)
**LL (n=45)**	46 (16-76)	21 (47%)	21 (47%)	3 (6%)
**BL (n=96)**	40 (14-78)	59 (61%)	34 (35%)	3 (3%)
**BB (n=24)**	40 (12-68)	19 (79%)	5 (21%)	0 (0%)
**BT (n=113)**	36 (8-74)	67 (59%)	42 (37%)	4 (4%)
**TT (n=131)**	24 (6-82)	76 (58%)	55 (42%)	0 (0%)

Supplementary Table S3: Leprosy classification, PGL-I antibody status, age, and gender distribution of the Brazilian cohort. LL = lepromatous leprosy; BL = borderline lepromatous; BT = borderline tuberculoid; M = male; F = female; NA = not available. PGL-I antibody (Ab) status: seronegative (IgM-/IgG-), IgM positive only (IgM+), IgG positive only (IgG+), or double positive (IgM+/IgG-).

	**Characteristics**				**PGL-I Ab status**			
	Median age (range)	M (%)	F (%)		IgM-/IgG-	IgM+	IgG+	IgM+/IgG+
**All patients (n=55)**	47 (12-78)	37 (62%)	23 (38%)		31(56%)	17 (31%)	0 (0%)	7 (13%)
**LL (n=17)**	47 (12-70)	15 (88%)	2 (12%)		7 (41%)	6 (35%)	0 (0%)	4 (24%)
**BL (n=12)**	50 (22-72)	6 (50%)	6 (50%)		4 (33%)	6 (50%)	0 (0%)	2(17%)
**BT (n=26)**	53 (18-78)	15 (58%)	11 (42%)		20 (77%)	5 (19%)	0 (0%)	1 (4%)
**Controls (n=27)**	30 (21-60)	10 (37%)	17 (63%)		27 (96%)	1 (4%)	0 (0%)	0 (0%)
